# Icaritin Improves Memory and Learning Ability by Decreasing BACE-1 Expression and the Bax/Bcl-2 Ratio in Senescence-Accelerated Mouse Prone 8 (SAMP8) Mice

**DOI:** 10.1155/2020/8963845

**Published:** 2020-06-29

**Authors:** Yuan-Yuan Li, Nan-Qu Huang, Fei Feng, Ying Li, Xiu-Mei Luo, Lin Tu, Jing-Qiu Qu, Yi-Man Xie, Yong Luo

**Affiliations:** ^1^Drug Clinical Trial Institution, The Third Affiliated Hospital of Zunyi Medical University (The First People's Hospital of Zunyi), Zunyi, Guizhou, China; ^2^Department of Neurology, The Third Affiliated Hospital of Zunyi Medical University (The First People's Hospital of Zunyi), Zunyi, Guizhou, China; ^3^Vascular Ultrasound Diagnostic Department, The Third Affiliated Hospital of Zunyi Medical University (The First People's Hospital of Zunyi), Zunyi, Guizhou, China

## Abstract

Icaritin (ICT) is the main component in the traditional Chinese herb *Epimedium*, and it has been shown to have anti-Alzheimer's disease (AD) effects, but its neuroprotective effects and the pharmacological mechanisms are unclear. In the present study, senescence-accelerated mouse prone 8 (SAMP8) mice were randomly divided into a model group and an ICT-treated group. Learning and memory abilities were detected by the Morris water maze assay, and the expression of amyloid beta protein (A*β*) and *β*-site APP cleavage enzyme 1 (BACE1) was determined by Western blotting and polymerase chain reaction (PCR). Histological changes in CA1 and CA3 were detected by hematoxylin-eosin staining (H&E staining), and the immunohistochemical analysis was used to detect the expression and localization of Bax and Bcl-2. The results showed that compared with the SAMP8 mice, the ICT-treated SAMP8 mice showed improvements in spatial learning and memory retention. In addition, the number of necrotic cells and the morphological changes in CA1 and CA3 areas were significantly alleviated in the group of ICT-treated SAMP8 mice, and the expression of BACE1, A*β*_1-42_ levels, and the Bax/Bcl-2 ratio in the hippocampus was obviously decreased in the ICT-treated group compared with the control group. The results demonstrated that ICT reduced BACE-1 levels, the contents of A*β*_1-42,_ and the Bax/Bcl-2 ratio, suggesting that ICT might have potential therapeutic benefits by delaying or modifying the progression of AD.

## 1. Introduction

Alzheimer's disease (AD) is the most common neurodegenerative disease and the most common cause of dementia. There are currently 47 million people worldwide suffering from dementia. This number is expected to increase to 131 million by 2050, increasing the burden on patients and their families and the burden on health and social care systems [1]. Understanding the cognitive and behavioral manifestations of dementia and their relationships to underlying brain pathology is one of the great challenges that neuropsychologists have faced in the past 50 years [1–3]. The aim of this study was to investigate the protective effects of icaritin (ICT) on learning and memory in a mouse model of AD, senescent-accelerated mouse-susceptible 8 (SAMP8) mice. There is no effective treatment for AD for a long time. The drugs currently used in treating AD mainly include acetylcholinesterase inhibitors, N-methyl-D-aspartate receptor antagonists, anti-A*β* drugs, and anti-inflammatory drugs. Although they have different mechanisms of action, these treatments have not achieved obvious therapeutic effects of AD [4].

To date, the most frequently cited AD pathological hallmark is the deposition of *β*-amyloid (A*β*). In recent years, the key focus of AD research has been the role of the secretase pathway in amyloid precursor protein (APP) cleavage to form A*β* [5, 6]. A*β* is produced from APP by *β*-site APP cleavage enzyme 1 (BACE1). Although BACE1 inhibitors are currently being used in clinical trials to treat AD patients, it remains critical to understand whether BACE1 inhibition significantly affects cognitive function in AD patients [7–9].

**Figure 1:**
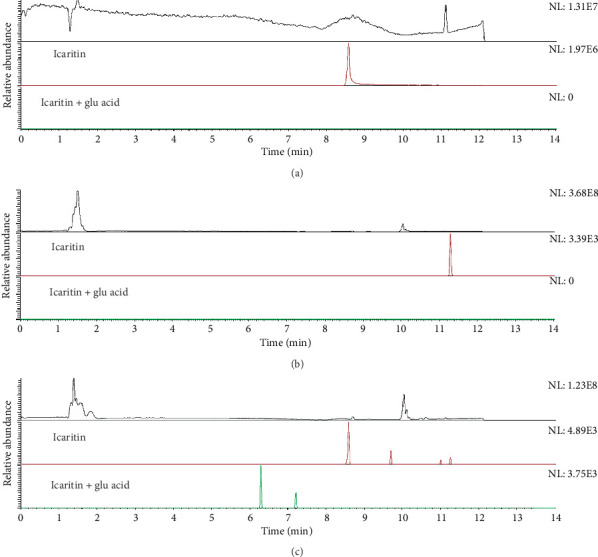
Detection of ICT in brain of rat by LC-MS. (a) Icaritin reference standard. (b) Brain of the control group, (c) Brain of the treatment group.

*Epimedium* is abundant in Guizhou, and it is a commonly used traditional Chinese medicine and ethnic medicine. It is rich in flavonoids. ICT (C_21_H_22_O_7_, MW: 386.4) and icaritin (ICA, C_33_H_40_O_15_, MW: 676.67) are active ingredients of flavonoids extracted from *Epimedium*. Our previous studies have shown that ICT exerts anti-AD effects by activating adenosine 5′-monophosphate-(AMP-) activated protein kinase (AMPK) and inhibiting BACE-1 [10]. However, the use of ICA presents some problems. First, ICA has poor solubility and poor absorption, resulting in low bioavailability. Second, ICA has an unstable chemical structure, and its preparation is difficult. Third, after administration of ICA, it is effectively unmeasurable in the brain. These factors make ICA itself unsuitable for the direct development of drugs. Fortunately, the molecular weight of ICT, a metabolite of ICA, is only 386.4 Da. The blood-brain barrier (BBB) is a microvascular barrier that strictly controls the passage of endogenous and exogenous molecules from blood to the central nervous system (CNS). However, a number of CNS diseases, such as brain tumors, nervous system trauma and stroke, degenerative diseases, and neurotoxic poisoning, have greatly increased the demand for effective therapeutic drugs, and it is particularly important to design drugs capable of passing the BBB [11–13]. In our previous study, we found that ICT can pass through the blood-brain barrier and be detected in the rat brain tissue after intragastric administration. The LC–MS results of ICT are shown in Figure 1. Therefore, we aimed to verify whether it also has an inhibitory effect on BACE-1 *in vivo* and the underlying mechanism.

Apoptosis theory is one of the important theories regarding the pathogenesis of AD, and the loss of a large number of neurons in the brain of AD patients is closely related to the mechanism of apoptosis [14]. A previous study found that DHA additive pretreatment might protect dopaminergic neurons in MPTP-induced mice by inhibiting apoptosis [15]. A*β* is a key etiology in AD, and targeting A*β* production and assembly is a new therapeutic strategy. Furthermore, investigators have shown A*β* to be toxic to neurons in both *in vitro* culture assays and the intact brains of animals [16]. A*β* can exert its cytotoxic effect by activating mitochondrial and endoplasmic reticulum pathways and A*β* protein-induced neuronal apoptosis, thereby causing oxidative stress and aggravating apoptosis [17]. Bax and Bcl-2 are indispensable in apoptotic cells and play opposite roles, determining whether cells survive or die. Bcl-2 is an antiapoptotic gene, the function of which is achieved by antioxidation, the inhibition of proapoptotic protein release, and the inhibition of Bax cytotoxicity and other mechanisms. The Bax/Bcl-2 ratio is considered to be one of the standards for evaluating apoptosis. Regulating the gene expression of Bax and Bcl-2 is important for inhibiting neuronal apoptosis [18–21].

In this study, we evaluated the effects of ICT on spatial learning and memory impairment in SAMP8 mice. Furthermore, we examined the effects of ICT on A*β* production and BACE1 expression and the expression of the apoptosis-related proteins Bax and Bcl-2.

## 2. Materials and Methods

### 2.1. Drugs and Chemicals

ICT (purity ≥ 98%) with a molecular weight of 68.38 g/mol (C_21_H_20_O_6_) was purchased from Aladdin Trading Co. Ltd. (Shanghai, China). All reagents were reagent grade and commercially available.

### 2.2. Animals and Drug Administration

In our study, male SAMP8 mice (5 months old) were purchased from the Peking University of Medicine (SPF-grade, certificate NO. SCXK (JING) 2016-0010), and SAMR1 mice were purchased from the Qinglong Mountain Animal Breeding Center of Nanjing (SCXK (Su) 2017-0001). The mice were housed in SPF-grade animal facilities (certificate NO. SYXK 2011-04) of Zunyi Medical University under a controlled ambient temperature (22°C–23°C) and humidity (50%–60%) and a 12 h light/dark cycle (lights on from 07 : 00 to 19 : 00). All animals in this study were allowed free access to food and water. The mice were used in the experiment once they reached 6 months of age.

The mice were randomly assigned to the following three groups (*n* = 5): a SAMR1 group, a SAMP8 group, and a SAMP8 + ICT group. All animal procedures followed the NIH guide for the Care and Use of Laboratory Animals (NIH Publications No. 80-23, revised 1978) and were approved by the Institutional Animal Use and Care Committee of Zunyi Medical College.

The mice in the SAMP8 + ICT group received daily gavages of 75 mg/kg ICT (5 mg/ml), while the mice in the SAMR1 and SAMP8 groups were intragastrically administered the same volume of normal saline for 22 days.

### 2.3. Morris Water Maze Task

**Figure 2:**
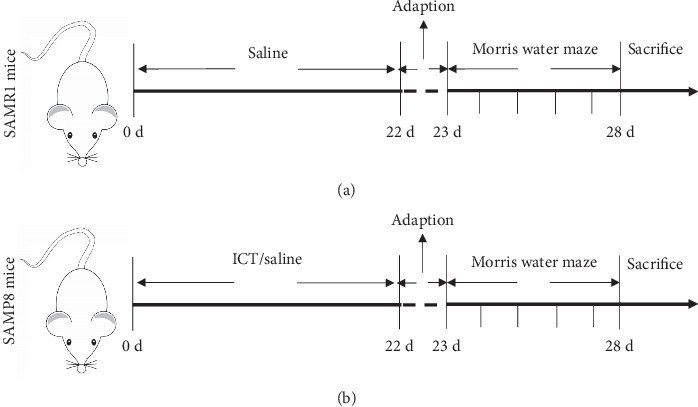
Design of the animal experiment.

The Morris water maze assay was applied to SAMR1 mice, SAMP8 mice, and ICT-treated SAMP8 mice 22 days after the initiation of ICT intervention to assess spatial learning and memory function, as shown in Figure 2. The formal experiment was conducted from the 23^rd^ day to the 27^th^ day after the initiation of ICT intervention. The directional navigation test: before this test, the indoor lights were turned off, and the mice were allowed to acclimate without disruption. One day before the experiment, each mouse was placed in a water maze without a platform and allowed to swim for 2 min to adapt to the swimming environment. At the beginning of the test, each mouse was slowly placed in the middle of the pool and encountered the water. The mouse was allotted 2 min to find the platform after entering the water; if it did not do so within 2 min, it was manually placed on the platform and allowed to remain there for 30 s. After the mice were fixed into the water from the four limits, the escape latency was recorded within 2 min. The average escape latency of each mouse was calculated as the daily learning ability index. The spatial learning experiment was carried out on the 28^th^ day. The platform was removed, and video of the mice swimming for 2 min was recorded using a TopScan^™^ behavioral testing system (version 3.0). Analysis was performed using the software analysis system.

### 2.4. Hematoxylin and Eosin Staining

Hippocampal slices were taken and fixed in 4% formalin for ≥72 h at 4°C, dehydrated in a gradient alcohol series, and embedded in paraffin. Each embedded sample was cut into 5 *μ*m sections on a rotary microtome. The sections were mounted on slides coated with 3-aminopropyltriethoxysilane and stained with H&E for 20 min at 4°C, followed by optical microscopy. Histopathological changes in the hippocampal CA1 and CA3 regions were observed and imaged using an Olympus microscope (BX60, Olympus, Japan).

### 2.5. Immunohistochemical Staining

Hippocampus tissue was sampled to perform immunohistochemical staining. The tissue samples were fixed with formaldehyde (10%) for 24 h and then embedded in paraffin before being cut into 5 *μ*m sections. Hippocampus tissues were immersed in PBS containing 4% (v/v) paraformaldehyde for 30 min and then dehydrated and embedded in paraffin. Sections of the specimens were made, and care was taken to prevent overstretching. Slides were incubated overnight at 4°C with the primary antibodies Bax (Abcam, Cambridge, UK) at a dilution of 1 : 300 and Bcl-2 (Abcam, Cambridge, UK) at a dilution of 1 : 100. After washing the sections with PBS, they were incubated with horseradish peroxidase-conjugated goat anti-rabbit IgG antibody as the secondary antibody at a dilution of 1 : 500 for 1 h at room temperature. The sections were then photographed using light microscopy (Olympus, Japan).

### 2.6. Western Blotting Analysis

Frozen hippocampus was homogenized in RIPA buffer supplemented with a protease inhibitor cocktail and centrifuged at 12,000 × g for 20 min at 4°C. The supernatants were collected, and total protein levels were quantified using the BCA protein assay kit (Beyotime, Shanghai, China). A total of 30 *μ*g of each sample was separated by 10% SDS-polyacrylamide gel. After electrophoresis, the proteins were transferred to a nitrocellulose membrane on ice at 100 V for 1 h. The membrane was blocked with 5% w/v skim milk powder in tris buffered saline (TBS-T) containing 0.05% Tween 20 for 1 h. After blocking, the membrane was incubated with a primary antibody overnight at 4°C. The following primary antibodies were used: anti-A*β* (6E10, 1 : 1000, Covance), rabbit polyclonal anti-BACE1 (1 : 1000, Sigma), and anti-*β*-actin (1 : 5000, Sigma). Immunoreactive bands were detected with HRP-conjugated goat anti-rabbit IgG (1 : 2000, Sigma). The membrane was washed with TBS-T, and the immune complex was visualized using an enhanced chemiluminescence detection kit (Thermo Scientific, USA). The membrane signal was scanned using a FluorChem Scanner and quantified using NIH ImageJ software. These results were normalized to the level of *β*-actin expression and confirmed by three measurements on the same sample.

### 2.7. RNA Extraction and RT-PCR

The hippocampal tissues of the three groups were collected. Total RNA was extracted using TRIzol reagent (Invitrogen, USA) according to the manufacturer's instructions. Each pellet was washed with 1 ml of 75% ethanol. The RNA was dissolved in DEPC water and was reverse transcribed into cDNA using the TaqMan reverse transcription reagents kit (Applied Biosystems). The tissues from three mice per group were used for RNA extraction. Real-time PCR was performed on a real-time PCR system (Bio-Rad, USA) using the SYBR Green PCR master mix according to the manufacturer's instructions. Briefly, the PCR cycling conditions consisted of an initial polymerase activation step at 95°C for 10 min followed by 40 cycles of 95°C for 5 s and 60°C for 1 min. A melting curve program was automatically initiated immediately upon the completion of PCR, consisting of denaturation at 95°C for 15 min, 60°C for 1 min, and 95°C for 15 s. The primers used for real-time PCR are listed in Table 1. The data were analyzed according to the delta-delta Ct (ΔΔCT) method and normalized against *β*-actin expression for each sample.

### 2.8. Statistical Analysis

The results are expressed as the mean ± SD, and all experiments were repeated three times. Analysis of variance (ANOVA) and Student's *t*-test were conducted by SPSS 19.0 (IBM, Armonk, NY, USA) software. A value of *P* < 0.05 was considered statistically significant.

## 3. Results

### 3.1. ICT Improved the Memory Impairment in SAMP8 Mice

**Figure 3:**
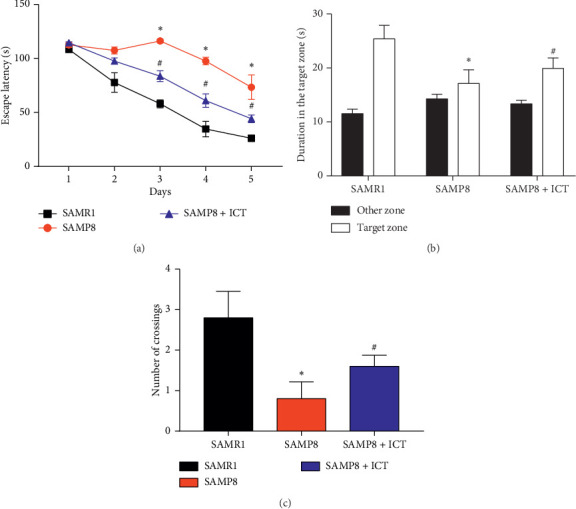
Effects of ICT on spatial learning and memory impairment in SAMP8 mice. (a) Effect of ICT on the escape latency of mice during five consecutive days of the hidden platform test. SAMP8 mice exhibited a longer escape latency in the training session than SAMR1 mice. ICT significantly reduced escape latency in SAMP8 mice. (b) Histograms showing the average swim time in the target quadrant and other quadrants during the probe test. (c) Comparisons of the number of platform crossings. Data are represented as the mean ± SD (*n* = 3), ^*∗*^*P* < 0.05*vs* the SAMR1 group, and ^#^*P* < 0.01*vs* the SAMP8 group.

The MWM test was performed to detect the effects of ICT on spatial learning and memory ability. The escape latency results during acquisition training are shown in Figure 3(a). The escape latency in the hidden platform acquisition phase showed a decreasing tendency with increasing training day. Compared to the SAMR1 mice, SAMP8 mice exhibited significantly longer escape latencies in the training session. ICT significantly shortened the escape latency in SAMP8 mice. After the training test, the probe test was performed to analyze the maintenance of memory. SAMP8 mice tended to exhibit less time in the target quadrant and more time spent in the other quadrants compared with SAMR1 mice, and ICT increased the time spent by SAMP8 mice in the target quadrant and decreased the time spent in other quadrants (Figure 3(b)). In addition, the number of crossings was significantly reduced in SAMP8 mice compared with SAMR1 mice, and ICT increased the number of crossings in SAMP8 mice (Figure 3(c)). Taken together, these results indicated that spatial learning and memory were impaired in SAMP8 mice and that ICT could improve the cognitive impairments.

### 3.2. ICT Treatment Inhibits BACE1 Expression and A*β* Production in SAMP8 Mouse Hippocampus

**Figure 4:**
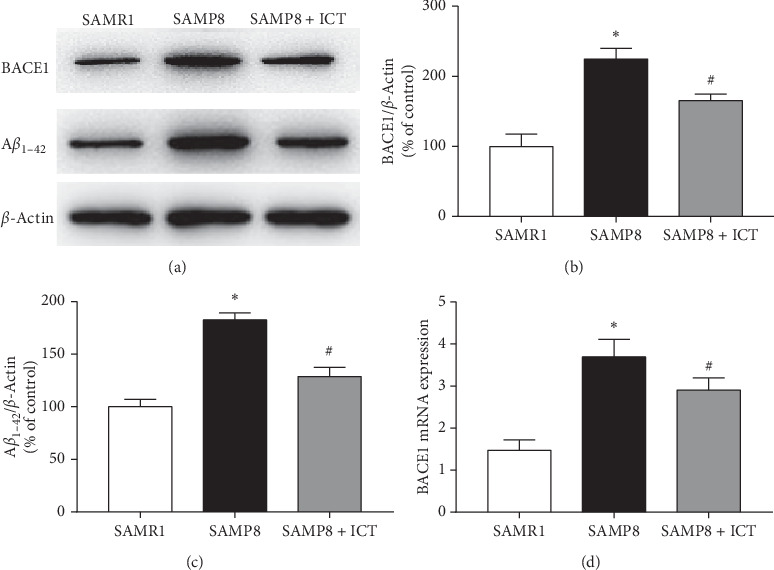
Effects of ICT on BACE1 and A*β*_1-42_ protein level in the hippocampus. (a) BACE1 and A*β*_1-42_ level in the hippocampus. (b), (c) Quantification of BACE1 and A*β*_1-42_. (d) BACE1 mRNA expression in the hippocampus. Values are mean ± SD (*n* = 3), ^*∗*^*P* < 0.05*vs* the SAMR1 group, and ^#^*P* < 0.01*vs* the SAMP8 group.

A*β*_1-42_ is produced by sequential cleavage of APP by BACE1. To determine whether changes in BACE1 protein expression were due to alterations in BACE1 transcription, we studied the ICT effect on BACE1 expression using a Western blot assay. As shown in Figures 4(a), 4(b), and 4(d), BACE1 expression levels were significantly increased in SAMP8 mice compared to SAMR1 mice (*P* < 0.01). ICT treatment greatly inhibited high levels of BACE1 expression in SAMP8 mice (*P* < 0.05) (Figures 4(a), 4(b), and 4(d)), suggesting that the decreased accumulation of A*β*_1-42_ may be due to the inhibitory effect of ICT on BACE1. A*β*_1-42_ is the main pathological marker for the development of AD. To investigate the effects of ICT on A*β* production, we determined the content of A*β*_1-42_ in the brain tissue by Western blot assay. Western blot results showed that the amount of A*β*_1-42_ deposited in SAMP8 mouse tissues (cortex and hippocampus) was significantly increased relative to the amount in SAMR1 mice (*P* < 0.05), while ICT was administered intragastrically for 22 days (with saline administration). Compared with SAMP8 mice, SAMP8 + ICA mice exhibited significantly reduced A*β*_1-42_ content (*P* < 0.05, Figures 4(a) and 4(c)).

### 3.3. Hematoxylin and Eosin Staining

**Figure 5:**
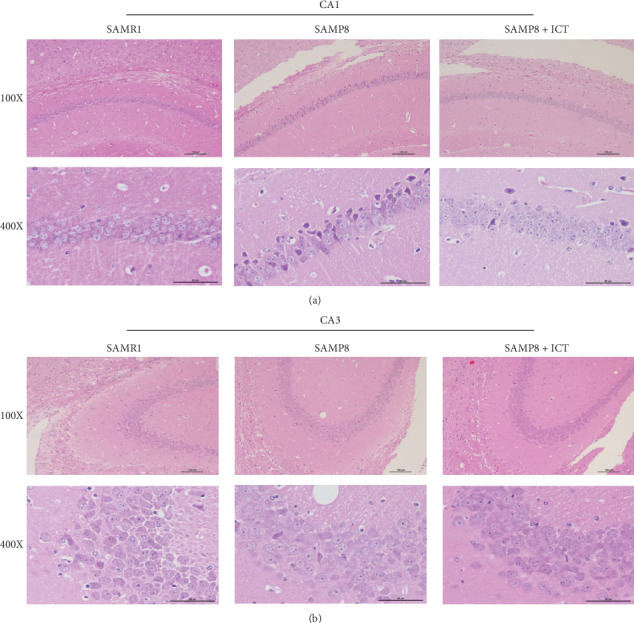
Effects of ICT on morphological alterations in the hippocampal CA1 and CA3 regions of SAMP8 mice (magnification, 100× and 400×). Representative sections were stained using HE. Compared with the SAMP8 group, the ICT treatment group exhibited a gradual improvement in condensed nuclei in the hippocampal CA1 and CA3 regions.

Brain tissues in each group were sectioned, and the pathological changes in the hippocampal region were assessed by H&E. As shown in Figure 5, H&E staining revealed unhealthy neurons in the CA1 and CA3 regions of the hippocampus of SAMP8 mice. Furthermore, most of the vertebral cell layer structure was disordered in SAMP8 mice, and the neurons appeared indistinct, with small, darkened nuclei. In contrast, few unhealthy cells were observed in the treatment groups compared with the SAMR1 group. However, neuropathological changes, including neuronal atrophy in the CA1 and CA3 regions of the hippocampus, were significantly ameliorated in the SAMP8 group, indicating that ICT can reduce neuronal injury in SAMP8 mice.

### 3.4. Icaritin Inhibited Neurons Apoptosis in SAMP8 Mice by Increasing the Bax/Bcl-2 Ratio

**Figure 6:**
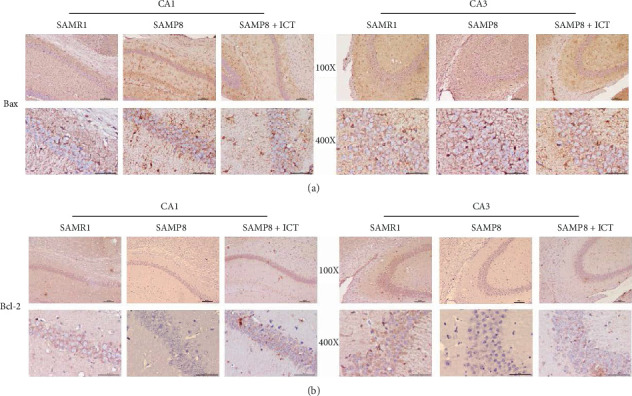
Bax and Bcl-2 expression.

**Figure 7:**
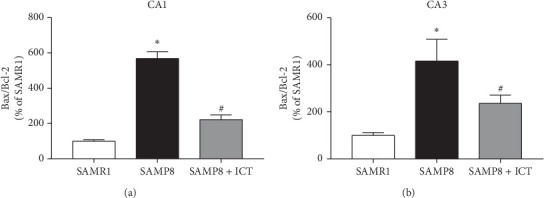
The Bax/Bcl-2 ratio in the CA1 and CA3 regions as determined by the immunohistochemistry assay. ^*∗*^*P* < 0.01 versus the SAMR1 group and ^#^*P* < 0.05 versus the SAMP8 group (*n* = 3).

Accumulating evidence indicates a close relationship between Bax/Bcl-2 level and diseases associated with apoptosis. The apoptosis-related proteins Bcl-2 and Bax are members of the Bcl-2 protein family. Bcl-2 is the primary protein that inhibits cell apoptosis and is expressed in healthy cells, whereas Bax promotes apoptosis. The ratio of Bax/Bcl-2 is considered a critical factor influencing apoptosis. To explore the mechanisms of antiapoptotic effects of ICT in SAMP8 mice, the levels of Bax and Bcl-2 were measured by immunohistochemistry. As shown in Figures 6(a) and 6(b), Bax expression was significantly increased in both the CA1 and CA3 regions of SAMP8 mice compared with the corresponding levels in the control group, and administration of ICT diminished as these increased. Furthermore, the expression of the antiapoptosis protein Bcl-2 was promoted by the apoptosis process in both the SAMP8 and SAMP8 + ICA groups. Regarding the Bcl-2/Bax ratio, in the SAMP8 group, the expression of Bcl-2 was decreased while that of Bax was increased, decreasing the Bcl-2/Bax ratio and indicating an imbalance between these proteins, as shown in Figures 7(a) and 7(b). The Bcl-2/Bax ratio was significantly higher in the SAMP8 + ICT group than in the SAMR1 group (*P* < 0.01) and the SAMP8 group (*P* < 0.05). The findings demonstrated that treatment with ICT diminished the increase in Bax expression and enhanced the expression of Bcl-2, leading to an increase in the Bcl-2 to Bax ratio. These results indicated that ICT may inhibit neuron apoptosis in SAMP8 mice by increasing the Bax/Bcl-2 ratio in the hippocampus.

## 4. Discussion

The present study demonstrated that ICT treatment improves spatial memory in a SAMP8 mouse model of AD. Furthermore, it showed that ICT treatment reduces A*β* production and BACE1 expression in the hippocampus of SAMP8 mice. Our data support the view that ICT is effective for the treatment of AD in mice. SAMP8 mice are rapid aging models characterized by brain aging. SAMR1 mice have the same genetic background as SAMP8 mice and are a typical antiaging model. As a model animal of aging, SAMP8 mice are excellent in simulating aging phenotypes, such as learning and memory disorders, massive deposition of A*β*, and typical AD pathological features, which can form A*β* deposits in the brain. The amount of A*β* deposition increases with age in these mice [22, 23]. SAMP8 mice are currently recognized as an AD model mice, which are characterized by mild cognitive dysfunction at 5 months of age and aggravation at 7 months of age [24]. Learning and memory dysfunction is the main feature of brain aging. The Morris water maze test can be used to evaluate spatial learning and memory in mice. This study used this behavioral evaluation method to test the learning and memory functions of SAMP8 mice. The results showed that 6-month-old SAMP8 mice have learning and memory dysfunctions and that ICT can improve these dysfunctions.

Studies have shown that the loss of learning and memory in SAMP8 mice can be attributed to early neurological damage in the mouse. Neurons are the basic functional units of the brain. Neuronal damage leads to declines in learning and memory. The more normal are the neurons, the better the brain can control learning and memory and other functional activities [25, 26]. H&E staining results showed that hippocampal neurons in aging mice were damaged, vertebral cells were disordered, and the number of normal cells was reduced. ICT can reduce neuronal degeneration, align vertebral cells, and increase the number of normal neurons. A*β* aggregation is one of the important factors leading to neuronal degradation. Decreasing the aggregation of A*β* is beneficial for improving the learning and memory functions of SAMP8 mice, reducing neuronal damage, and delaying aging. The accumulation of A*β* reflects a loss of the dynamic balance of A*β* levels in the brain, with increased A*β* production and reduced clearance or degradation. The precursor protein of A*β* and APP can be metabolized by *β*-secretase and *γ*-secretase to produce A*β*. *β*-Secretase is the rate-limiting enzyme in the metabolism of APP to produce A*β*. BACE1 has the biological activity of *β*-secretase and reduces the expression of BACE1 protein. Inhibition of the *β*-secretase activity reduces A*β* production [27–30]. The results of this experiment show that ICT can downregulate the protein level of BACE1 and play a neuroprotective role by reducing A*β* production. A*β* accumulation was proved to be able to lead to neuronal apoptosis, which was regulated by some regulators, such as the antiapoptotic Bcl-2 and proapoptotic Bax. It has been reported that the relative expression of Bax/Bcl-2 was significantly increased in A*β*_1-42_-injected rats compared with that of the sham group rats, and neuronal apoptosis will be caused by intracerebroventricular injection of A*β*_1-42_ [31, 32]. Apoptosis is the main reason for the loss of dopaminergic neurons. When apoptosis occurs, Bax forms a homodimer and migrates into the cytoplasm. Located in the mitochondria, it alters the permeability of the mitochondrial membrane, thereby promoting cytochrome c (cyt c), which is an apoptosis inducing factor. When cyt c is released and Bcl-2 family member Bax forms a heterodimer with Bcl-2, Bcl-2 plays a role in inhibiting apoptosis. Wang et al. found that PD symptoms in mice will be relieved by inhibiting the apoptosis via a mitochondria-mediated pathway [33]. So, Western blot was used to detect the extent of apoptosis and the expression of the antiapoptotic proteins Bax and Bcl-2 in the brain tissue. These two proteins mainly regulate the apoptotic pathway. The results show that ICT can effectively reduce the Bax/Bcl-2 ratio. We speculate that ICT may protect neurons from damage through its antiapoptotic effects.

## 5. Conclusion

In summary, the current study demonstrated that ICT treatment reduced A*β* production by downregulating the expression of BACE1 in the hippocampus of SAMP8 mice. ICT increases the Bcl-2/Bax ratio to inhibit the neuronal apoptosis response, thus improving memory and learning abilities. These novel findings suggest that ICT treatment may have potential for blocking or delaying pathological progression.

## Figures and Tables

**Table 1:** Primer sequences used for the amplification of target genes.

Gene	Forward 5′–3′	Reverse 5′–3′
BACE1	GTATCGACCACTCGCTATACAC	GTACTCCTTGCAGTCCATCTT
*β*-actin	CTCCCTGGAGAAGAGCTATGA	CCAAGAAGGAAGGCTGGAAA

## Data Availability

The data used to support the findings of this study are available from the corresponding author upon request.
